# Impacts of surface wear of attachments on maxillary canine distalization with clear aligners: a three-dimensional finite element study

**DOI:** 10.3389/fbioe.2025.1530133

**Published:** 2025-01-21

**Authors:** Qiuying Li, Bowen Xu, Dongyu Fang, Kai Yang

**Affiliations:** Department of Orthodontics, School of Stomatology, Capital Medical University, Beijing, China

**Keywords:** wear, attachment, clear aligner, canine distalization, finite element study

## Abstract

**Objectives:**

This study established three-dimensional finite element models to explore the impacts of surface wear of attachments on maxillary canine distalization with clear aligners, thereby guiding the clinical application of attachments and enhancing the efficiency of clear aligner therapy.

**Materials and methods:**

Finite element models of maxillary canine distalization, including the maxilla, dentition, periodontal ligament, attachments (in both initial and worn states), and clear aligners, were established. Two groups of attachments (vertical rectangular attachment and optimized root control attachment) and five working conditions representing different degrees of attachment wear (M0, M2, M4, M6, and M8) were designed for canine distalization. Tooth displacement and equivalent stress in the roots and periodontal ligaments were analyzed.

**Results:**

The canines in both groups exhibited a tipping movement pattern under all working conditions. By M8, the distal displacement of the canine crown, the equivalent stress values in the roots, and the equivalent stress values in the periodontal ligaments in the rectangular attachment group decreased by 12.04%, 30.80%, and 16.48%, respectively, compared to M0. In the optimized root control attachment group, these values decreased by 24.98%, 34.69%, and 19.15%, respectively. However, under all working conditions, the canines in the rectangular attachment group presented greater displacement and stress. The greatest reduction in canine crown distal displacement and stress values was observed between M6 and M8 in the rectangular attachment group, but the efficiency of canine distalization was still 64.30% at M8, with minimal change. In the optimized root control attachment group, the greatest reduction was observed in M4–M6, and the efficiency of canine distalization decreased to less than 60% in response to M6.

**Conclusion:**

The canines tended to tip when maxillary canine distalization was performed with clear aligners. Attachment wear led to a reduction in the efficiency of canine distalization. Compared with optimized root control attachments, the impact was less significant for rectangular attachments. Once optimized root control attachments have been in place for more than 4 months and maxillary canine distalization is still required, orthodontists should closely monitor the wear of these attachments. If necessary, timely restoration or rebonding of the attachments is recommended.

## 1 Introduction

With rapid advancements in biomaterials, computer-aided design and manufacturing, clear aligner therapy has emerged as a promising alternative to conventional fixed appliances in orthodontics (Bichu et al., 2023). A recent survey indicated that clear aligner therapy was provided by more than 90% of orthodontists (Meade and Weir, 2022). Compared with fixed appliances, clear aligners offer several advantages, including aesthetics, comfort, and ease of oral hygiene maintenance, thereby improving the quality of life of patients (Pithon et al., 2019; Li et al., 2023; Liu et al., 2024). Despite the numerous advantages of clear aligners, they still suffer from poor tooth control when performing complex tooth movements because of their material properties (Kravitz et al., 2009; Simon et al., 2014).

Attachments serve as crucial auxiliary devices in clear aligner therapy. They are composite resin structures bonded to the labial or lingual surfaces of the teeth, which enhance the retention of the aligners and assist in tooth movement, thereby improving treatment efficiency (Groody et al., 2023; Lear et al., 2024). Owing to brushing, eating, and frequent removal of aligners, surface wear may occur on the attachments. This phenomenon can impact the biomechanics of the clear aligner system, potentially affecting the efficacy of tooth movement (Mantovani et al., 2019). Several studies have focused on the surface wear of attachments in clear aligner therapy. Through a longitudinal study, Barreda et al. reported that attachment wear occurred after 6 months of aligner wear (Barreda et al., 2017). A recent study reported that higher levels of surface wear were observed in conventional attachments and those bonded to anterior and mandibular teeth (Fausto da Veiga Jardim et al., 2024). Our recent research quantitatively evaluated the surface wear of attachments and investigated the corresponding risk factors (Li and Yang, 2024). However, the exact impact of attachment wear on tooth movement has rarely been studied.

During clear aligner therapy, canine distalization often results in distal crown tipping rather than bodily movement. The movement pattern and position of the canines significantly influence treatment outcomes, leading many scholars to focus on the efficiency and treatment planning strategies for canine distalization with clear aligners. Some studies have reported that the addition of attachments can facilitate the bodily movement of canines and improve canine distalization efficiency (Gomez et al., 2015; Chen et al., 2024). Therefore, the wear of attachments may affect canine distalization outcomes.

Finite element analysis is a numerical method that has been widely used in oral medicine research, particularly in the field of orthodontics (Merdji et al., 2013; Duanmu et al., 2021; Kang et al., 2023; Koru Akan et al., 2024). These simulations effectively illustrate complex and biomechanical responses during orthodontic treatment, including tooth displacement and stress distribution (Benaissa et al., 2020; Chen et al., 2024; Ouldyerou et al., 2024). The biomechanisms of clear aligner therapy are not yet fully understood. Compared with *in vitro* experiments, the finite element method is currently the most common and convenient approach for studying these mechanisms.

This study established three-dimensional (3D) finite element models of maxillary canine distalization with clear aligners. On the basis of the results from our previous clinical trial, the models simulated attachment wear to explore its impact on maxillary canine distalization (Li and Yang, 2024). This study aimed to provide guidance for the clinical application of attachments, thereby enhancing the efficiency of clear aligner therapy.

## 2 Materials and methods

### 2.1 Construction of the 3D finite element model

This study was approved by the Research Ethics Committee of Beijing Stomatological Hospital, Capital Medical University (CMUSH-IRB-KJ-PJ-2022–25). An adult volunteer at Beijing Stomatological Hospital, Capital Medical University, was recruited for cone beam computed tomography (CBCT, NewTom VGi, Italy) data acquisition. A written informed consent form was obtained from the volunteer. The volunteer met the following criteria: individual normal occlusion, good dental anatomy, good periodontal condition, and no systemic or oral diseases.

Two groups of sets were designed in this study. Model #1: Horizontal rectangular attachments were positioned on the right maxillary canine. Model #2: The optimized root control attachments were positioned on the right maxillary canine. For both group sets, five working conditions representing different degrees of attachment wear were designed on the basis of findings from our previous clinical study (Li and Yang, 2024). The conditions M0, M2, M4, M6, and M8 correspond to the amount and location of attachment wear obtained in patients wearing clear aligners for 0, 2, 4, 6, and 8 months, respectively.

**FIGURE 1:**
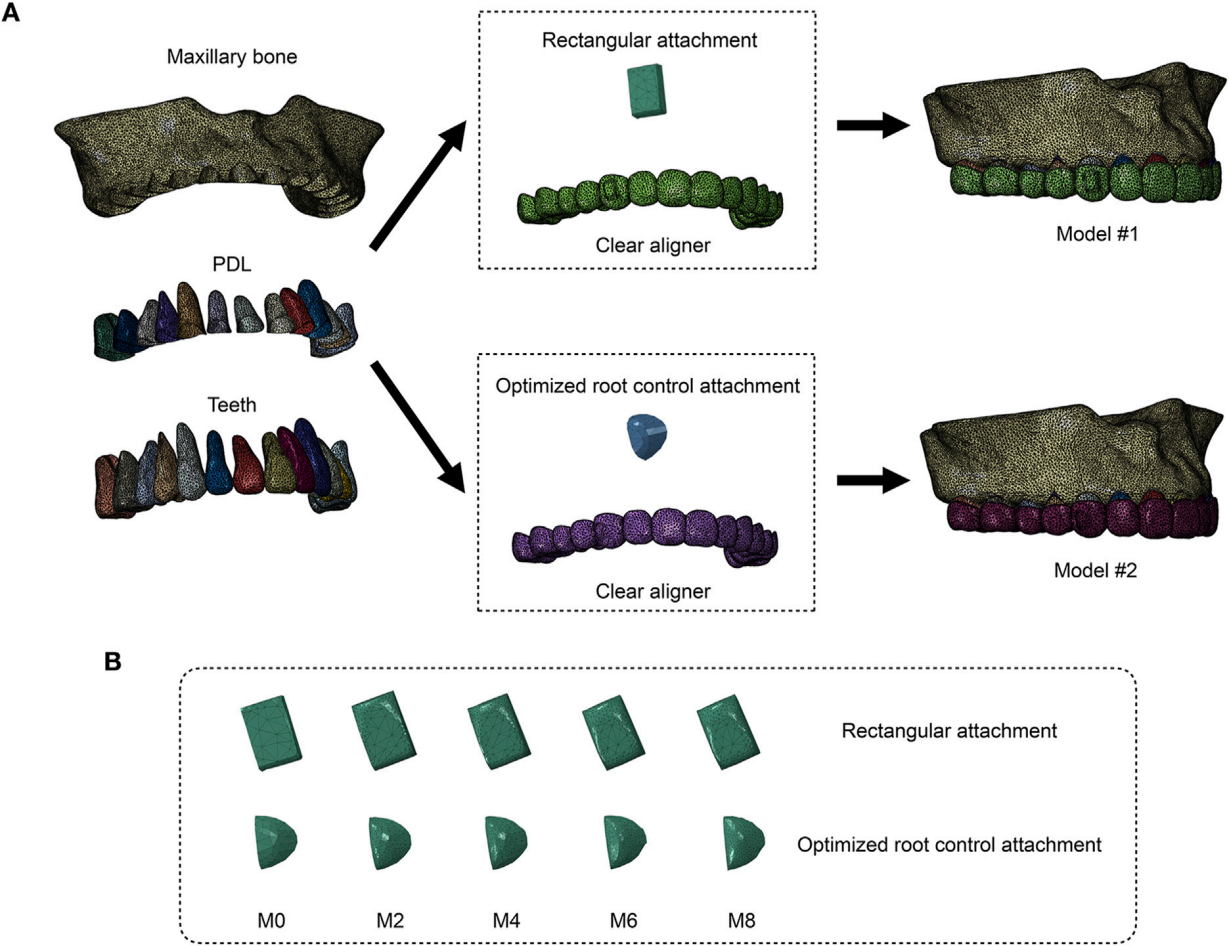
Two groups of models were constructed to simulate right maxillary canine distalization (Model #1: vertical rectangular attachment group; Model #2: optimized root control attachment group). **(A)** All the components of the finite element models. PDL: periodontal ligament. **(B)** Finite element models of attachments in different wear states. M0, M2, M4, M6, and M8 correspond to the amount and location of attachment wear obtained in patients wearing clear aligners for 0, 2, 4, 6, and 8 months, respectively.

The CBCT data were imported into Mimics 21.0 (Materialise, Belgium), where image segmentation was performed by adjusting thresholds on the basis of variations in tissue gray values. In this process, preliminary 3D models of the maxilla and dentition were constructed. The 3D models were exported in STL format and imported into Geomagic Studio 2014 (3D Systems Co., United States) for surface smoothing. The geometric models of the periodontal ligament (PDL), attachments, and clear aligners were constructed by using Pro/E 5.0 (Parametric, United States). PDL was created by extending 0.20 mm away from the root surface (Hong et al., 2021). The horizontal rectangular attachments were positioned at the center of the buccal surface of the right maxillary canine. The initial size of the rectangular attachments was set as 3 × 2 × 1 mm. The initial shape and location of the optimized root control attachments were designed on the basis of Clincheck (Align Tech, United States). Attachments after wear were constructed by performing a Boolean operation on initial attachments. The right maxillary canine with the initial attachment was distally moved by 0.2 mm. The aligner models were subsequently constructed by making an external offset of 0.75 mm thickness on the dentition and attachments (Fan et al., 2022). The components of each model (maxilla, dentition, PDL, attachments, clear aligners) were assembled and converted into a 3D finite element model with unstructured 4-noded tetrahedral elements in Hypermesh 14.0 (Altair, United States). The numbers of nodes and elements for all the components are summarized in Table 1. The models were imported into Abaqus 6.14 (Dassault Systems, France). The finite element models of all the components are shown in Figure 1.

**TABLE 1:** Number of elements and nodes for the finite element models.

Component	Model #1		Model #2	
	Elements	Nodes	Elements	Nodes
Maxilla	358,873	74,341	358,873	74,341
Teeth	70,856	20,656	70,856	20,656
PDL	61,621	19,995	61,621	19,995
Attachment				
*M0*	260	114	130	60
*M2*	1,453	492	850	283
*M4*	1,947	628	1,361	445
*M6*	1,705	559	1,492	488
*M8*	585	223	1,302	447
Clear aligner	57,857	17,949	60,043	18,587

Model #1: rectangular attachment group; Model #2: optimized root control attachment group; PDL: periodontal ligament.

### 2.2 Material properties, boundary constraints, and contact conditions

The material properties of all the components were set on the basis of previous studies (Liang et al., 2009; Gomez et al., 2015; Fang et al., 2020) (Table 2). The maxilla, teeth, PDL, attachment, and clear aligner were all defined as homogeneous, isotropic linear elastic materials. The upper part of the maxilla was set as a fixed constraint. Bonded contacts were set between the alveolar bone and the PDL, PDL and teeth, as well as between teeth and attachments (Li et al., 2024). Surface-to-surface contact was set between the internal surface of the clear aligner and the external surface of the teeth/attachments, with a friction coefficient of μ = 0.2 (Ramalho and Antunes, 2007). No additional constraints or loads were applied to the models.

**TABLE 2:** Material properties.

Material	Young’s modulus (MPa)	Poisson’s ratio
Maxilla	1.37 × 10^3^	0.30
Teeth	1.96 × 10^4^	0.30
PDL	0.67	0.45
Attachment	12.5 × 10^3^	0.36
Clear aligner	842	0.36

PDL: periodontal ligament.

### 2.3 Coordinate system setting

Two coordinate systems were established in this study. The global coordinate system was set on the maxilla. The x-, y-, and *z*-axes represent the sagittal, coronal, and vertical directions, respectively. To better evaluate and analyze the displacement of the right maxillary canine, a local coordinate system was established for the right maxillary canine, with the cusp of the canine as the origin. The *x*-axis was oriented in the sagittal direction, the *y*-axis was oriented in the coronal direction, and the *z*-axis was oriented in the vertical direction, with positive directions toward the distal side, the lingual side, and the crown, respectively.

### 2.4 Data analysis

The right maxillary canine was taken as the target tooth. The following parameters were analyzed in this study:

1) Displacement tendencies. 2) Distal displacement of the crown. The center of the labial surface of the canine crown was taken as the measuring point. 3) The equivalent stress in the roots. 4) The equivalent stress in the PDL.

To better analyze the impact of attachment wear on canine distalization, additional parameters were also calculated:

1) The rate of change in the crown distal displacement, defined as (the crown distal displacement at M0 − the crown distal displacement at Mn)/the crown distal displacement at M0 × 100%, with n values of 2, 4, 6 and 8.
2) Efficiency of canine distalization, defined as the distal displacement of the canine crown at Mn/0.2 × 100%, with n values of 0, 2, 4, 6 and 8.
3) The rate of change in the equivalent stress in the roots, defined as (the equivalent stress in the roots at M0 − the equivalent stress in the roots at Mn)/the equivalent stress in the roots at M0 × 100%, with n values of 2, 4, 6 and 8.
4) The rate of change in the equivalent stress in the PDL, defined as (the equivalent stress in the PDL at M0 − the equivalent stress in the PDL at Mn)/the equivalent stress in the PDL at M0 × 100%, with n values of 2, 4, 6 and 8.

## 3 Results

### 3.1 Initial displacement and displacement tendencies of the canine

**FIGURE 2:**
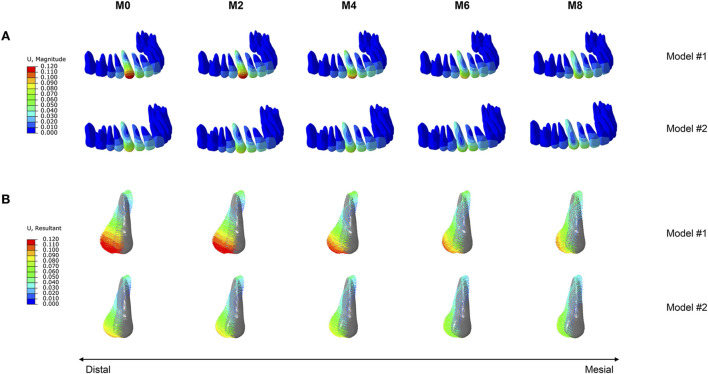
Tooth displacement in the two groups of models (Model #1: vertical rectangular attachment group; Model #2: optimized root control attachment group). **(A)** Dentition displacement. **(B)** Displacement tendencies of the canine. M0, M2, M4, M6, and M8 correspond to the amount and location of attachment wear obtained in patients wearing clear aligners for 0, 2, 4, 6, and 8 months, respectively.

**FIGURE 3:**
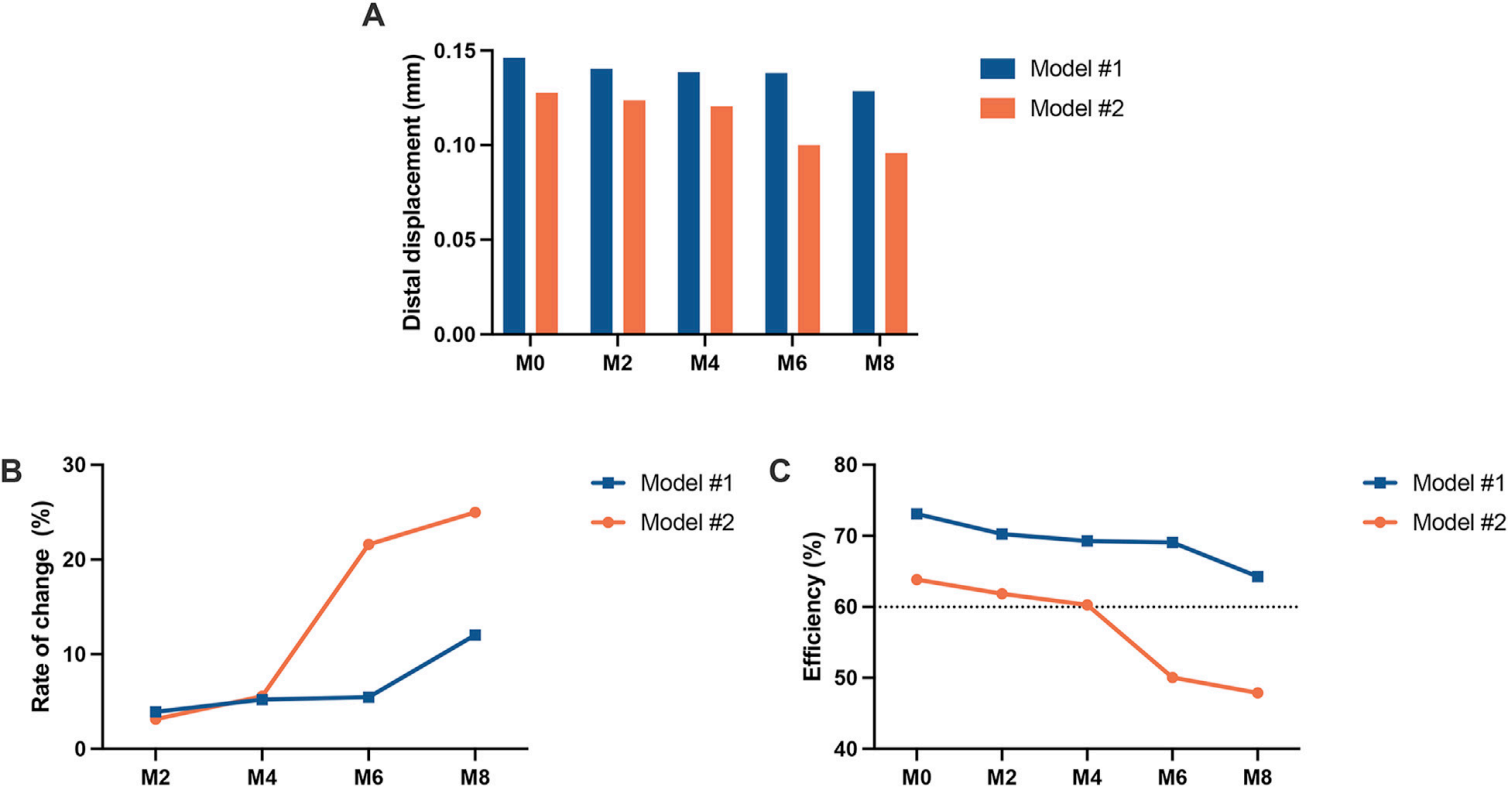
Displacement of the canine crown in the two groups of models (Model #1: vertical rectangular attachment group; Model #2: optimized root control attachment group). **(A)** Distal displacement of the crown. **(B)** The rate of change in crown distal displacement. **(C)** Efficiency of canine distalization. M0, M2, M4, M6, and M8 correspond to the amount and location of attachment wear obtained in patients wearing clear aligners for 0, 2, 4, 6, and 8 months, respectively.

The dentition displacement and displacement tendencies of the canine are shown in Figure 2. Under all working conditions, both groups of models exhibited a tipping trend, with the crown moving distally and the root moving mesially. Increasing attachment wear led to a decrease in the distal displacement of the canine crown in both groups. Moreover, Model #1 resulted in greater distal displacement than Model #2 across all working conditions (Figure 3A). In Model #1, the distal displacement of the canine crown decreased by 3.90% at M2 and 12.04% at M8 compared with M0. In contrast, Model #2 experienced decreases of 3.13% at M2 and 24.98% at M8. The greatest reduction in canine crown distal displacement was observed in Model #1 between M6 and M8, whereas Model #2 demonstrated the most notable decrease between M4 and M6 (Figure 3B). In Model #2, the efficiency of canine distalization declined below 60% by M6, whereas Model #1 maintained approximately 64% at M8 with minimal change (Figure 3C).

### 3.2 Equivalent stress in the roots

**FIGURE 4:**
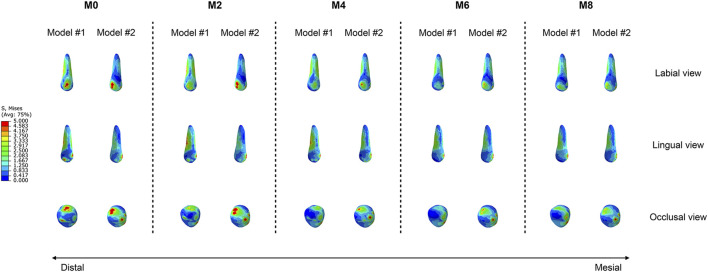
Equivalent stress distributions of the canines in the two groups of models (Model #1: vertical rectangular attachment group; Model #2: optimized root control attachment group). M0, M2, M4, M6, and M8 correspond to the amount and location of attachment wear obtained in patients wearing clear aligners for 0, 2, 4, 6, and 8 months, respectively.

**FIGURE 5:**
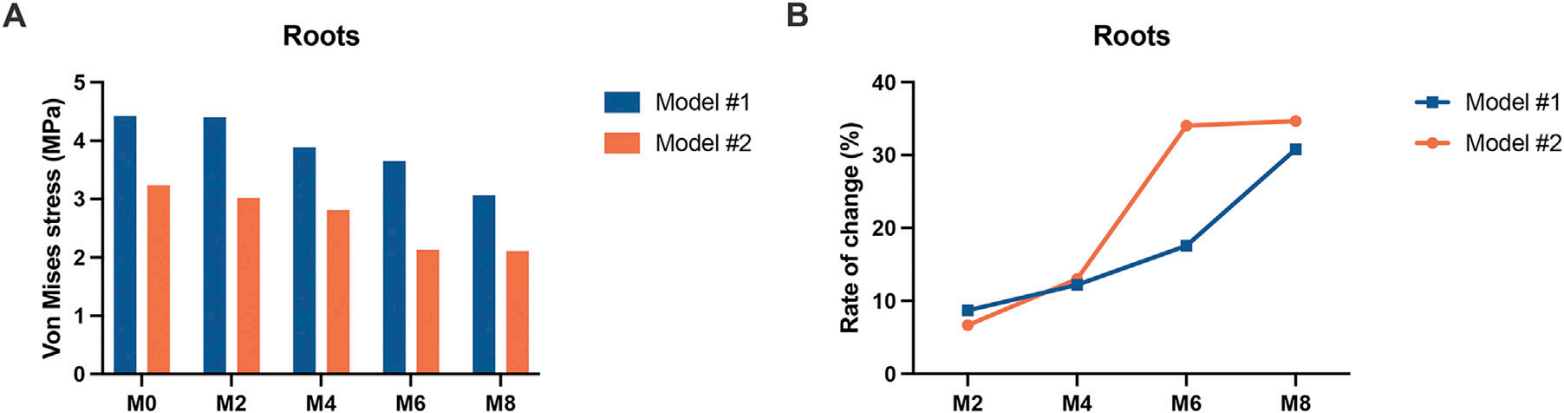
The equivalent stress in the canine roots of the two groups of models (Model #1: vertical rectangular attachment group; Model #2: optimized root control attachment group). **(A)** The equivalent stress values of the roots. **(B)** Rate of change in the equivalent stress in the roots. M0, M2, M4, M6, and M8 correspond to the amount and location of attachment wear obtained in patients wearing clear aligners for 0, 2, 4, 6, and 8 months, respectively.

The equivalent stress distribution of the canine is shown in Figure 4. At M0, both groups of models presented stress concentrations in the crown at the attachment bonding area, with root stress primarily concentrated on the mesiodistal surfaces, reaching a peak near the cervical region. As the degree of attachment wear increased, the von Mises stress values of the roots gradually decreased, whereas the pattern of stress distribution remained consistent. The root equivalent stress values of Model #1 were greater than those of Model #2 across all working conditions (Figure 5A). In Model #1, the root equivalent stress decreased by 8.72% at M2 and 30.80% at M8 compared with M0. In Model #2, the decreases were 6.68% at M2 and 34.69% at M8. The most significant reduction in root equivalent stress in Model #1 occurred between M6 and M8, whereas in Model #2, it was between M4 and M6 (Figure 5B).

### 3.3 Equivalent stress in the PDL

**FIGURE 6:**
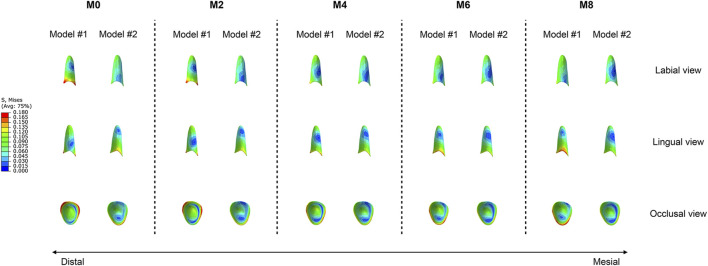
Equivalent stress distributions of the canine periodontal ligament in the two groups of models (Model #1: vertical rectangular attachment group; Model #2: optimized root control attachment group). M0, M2, M4, M6, and M8 correspond to the amount and location of attachment wear obtained in patients wearing clear aligners for 0, 2, 4, 6, and 8 months, respectively.

**FIGURE 7:**
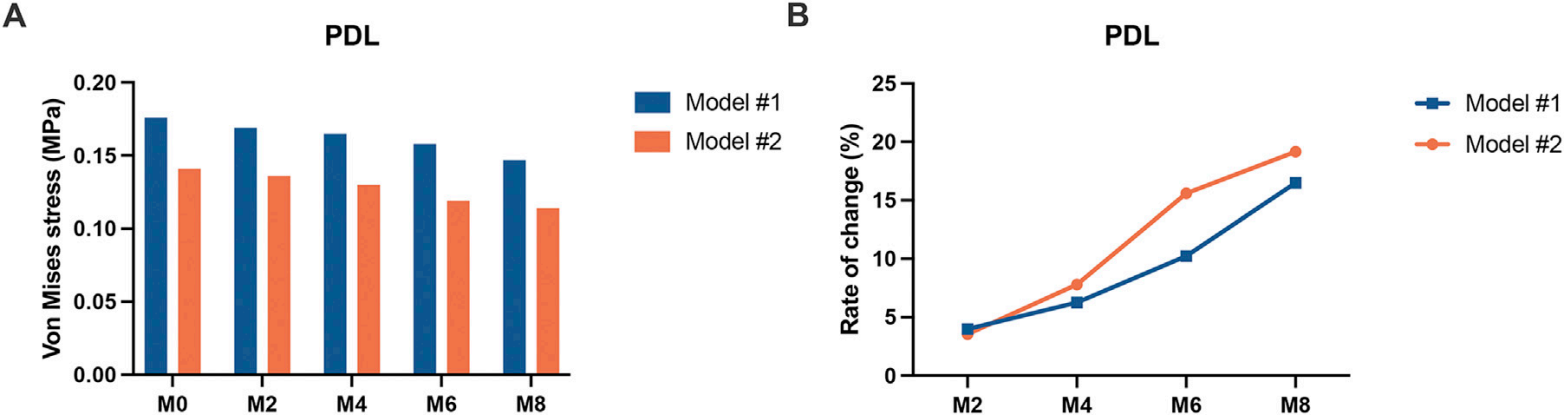
The equivalent stress in the canine periodontal ligament of the two groups of models (Model #1: vertical rectangular attachment group; Model #2: optimized root control attachment group). **(A)** The equivalent stress values of the periodontal ligament. **(B)** Rate of change in the equivalent stress in the periodontal ligament. PDL: periodontal ligament. M0, M2, M4, M6, and M8 correspond to the amount and location of attachment wear obtained in patients wearing clear aligners for 0, 2, 4, 6, and 8 months, respectively.

The equivalent stress distribution of the PDL is shown in Figure 6. As the wear of the attachments increased, the von Mises stress values in the PDL of both models gradually decreased. However, for Model 1, in the M0 condition, the region of maximum PDL stress was located at the labial cervical area, followed by the apical region. As the wear of the attachments increased, the region of maximum stress gradually shifted to the lingual cervical area, and the stress distribution in the apical region gradually decreased. For Model 2, in all conditions, the region of maximum PDL stress was consistently located at the lingual cervical area, and the range of maximum stress distribution also gradually decreased. The trend in the equivalent stress values of the PDL was similar to that of the roots. The PDL equivalent stress values of Model #1 were greater than those of Model #2 across all working conditions (Figure 7A). In Model #1, the PDL equivalent stress decreased by 3.98% at M2 and 16.48% at M8 compared with M0. In Model #2, the decreases were 3.55% at M2 and 19.15% at M8. The greatest reduction was shown between M6 and M8 for Model #1 and between M4 and M6 for Model #2 (Figure 7B).

## 4 Discussion

Traditional fixed orthodontic techniques generate forces through the deformation of archwires, which are transmitted to the teeth via brackets bonded to the tooth surfaces to control the tooth position. However, clear aligner techniques generate forces through the deformation of thermoplastic materials, which are transferred to the teeth via aligners that cover the tooth surfaces and subsequently transmitted to the tooth roots and periodontal tissues to achieve tooth movement. Therefore, the fit between the aligner and the tooth surface is crucial. This study revealed that as attachment wear increased, the distal displacement of the canine crown and the von Mises stress values of the roots and PDL decreased. Xu et al. also established a three-dimensional finite element model for maxillary canine distalization (Xu et al., 2018). They added power arms and vertical rectangular attachments to the maxillary canines, resulting in distalization efficiencies of 94% and 73%, respectively. Compared to their results, the distalization efficiency of the rectangular attachment group in our study, in the unworn state, is consistent with their findings. However, when the attachments are in a worn state, the distalization efficiency in our study is lower than their results. We attribute this result to the wear of the attachments, which reduces the fit between the aligner and the tooth surface. This decrease in fit leads to a reduction in the force transmitted from the aligner to the tooth. As the fit between the attachments and the aligner deteriorates, the efficiency of canine distalization also decreases. A previous clinical study showed that canines with rectangular attachments exhibited a certain degree of lingual tipping during distalization (Ren et al., 2022). The results of this study also indicate that as attachment wear increases, the region of maximum PDL stress in the rectangular attachment group shifts from the labial cervical area to the lingual cervical area. We speculate that one possible reason for this result is that attachment wear leads to a decrease in the fit between the aligner and the attachment, thereby reducing the aligner’s control over the tooth.

Our previous clinical study found that the wear of rectangular attachments began at the edges and gradually progressed towards the buccal surface, with the most significant wear occurring at the edges closest to the gingiva (Li and Yang, 2024). In contrast, the wear of optimized root control attachments was primarily concentrated on the marginal ridges of the surface. During clinical follow-up visits, orthodontists are recommended to closely monitor these areas to track attachment wear. Additionally, intraoral scans can be performed, and wear can be observed through three-dimensional superimposition analysis. On the basis of our study results, for the optimized root control attachments, when the wear was equivalent to 4 months of aligner wear, further increases in wear led to a significant decrease in the distal displacement of the canine crown and the von Mises stress values of the roots and PDL. When the wear was equivalent to 6 months of aligner wear, the efficiency of canine distalization decreased to less than 60%. If the optimized root control attachments have been in place for more than 4 months and maxillary canine distalization is still required, orthodontists should pay particular attention to the wear of these attachments to ensure the efficiency of canine distalization. If an attachment has been lost, it should be re-bonded using a template. If the attachment’s shape has significantly changed, it is recommended to remove the existing attachment and re-bond it using a template. If the attachment’s shape has not significantly changed but wear is evident, the attachment can remain in place, and holes can be made in the template to inject flowable resin and restore the attachment’s shape.

Owing to the low stiffness and rapid force deflection of thermoplastic materials, clear aligners often fail to fully achieve the designed movement. This discrepancy between the designed and actual movements has been analyzed by several scholars. Castroflorio et al. reported that for every 1 mm of planned maxillary canine distalization, 0.4 mm was lost (Castroflorio et al., 2023). Our study revealed similar results, with the efficiency of canine distalization across all conditions ranging from 48% to 73%. Under all working conditions, the distal displacement of the canine crown and the von Mises stress values of the roots and PDL were greater with rectangular attachments than with optimized root control attachments. This finding indicates that even with wear, the addition of rectangular attachments could achieve greater efficiency in maxillary canine distalization. Furthermore, Ren et al. demonstrated that in cases of clear aligner therapy involving the extraction of maxillary first premolars, adding vertical rectangular attachments to canines resulted in more predictable incisor movement than optimized attachments (Ren et al., 2022). Therefore, based on our findings, considering the impact of attachment wear on tooth movement, we recommend that orthodontists design vertical rectangular attachments on the maxillary canines to improve the efficiency of canine distalization during clear aligner treatment.

The results of this study also demonstrate that regardless of the type of attachment added to the canines or the amount of attachment wear, the canines display a tipping movement. These findings align with those of previous studies. Ho et al. used a typodont model to simulate canine distalization *in vitro* and reported that even with attachments, the canines exhibited tipping movement during distalization (Ho et al., 2021). Chen et al. reached the same conclusion (Chen et al., 2024). In this study, the maximum stress in the roots and PDL was primarily distributed in the cervical region under all working conditions. This distribution pattern indicates that the orthodontic forces generated by clear aligners are applied mainly to the clinical crown rather than the center of the tooth, resulting in crown tipping rather than bodily movement. This suggests that in clinical treatment, if bodily movement of the canine is desired, adding attachments alone is inadequate. Additional measures, such as adding torque to the canines in the treatment plan or using power arms during treatment, should be considered (Inan and Gonca, 2023).

The foundation of finite element analysis is the establishment of the model. The accuracy and scientific validity of the finite element results are largely determined by the precision of the model. This study used CBCT data in DICOM format for modeling, which provides a relatively high level of precision, facilitating more accurate finite element analysis results (Nagasao et al., 2002). Furthermore, based on our previous clinical trials, we obtained the wear amounts and primary wear locations of the two most commonly used attachments during canine distalization. The wear attachment models were established using Boolean operations based on the statistical analysis results, enhancing their generalizability. Additionally, a full maxillary dental arch model was constructed, which better reflects clinical reality. However, there are certain limitations in the model establishment process of this study. First, the finite element models established in this study, similar to most current research, are static (Guo et al., 2024; Yang et al., 2024). They can provide only immediate displacement and stress values after the load is applied, making accurate simulation of the dynamic process of alveolar bone remodeling that occurs during orthodontic treatment difficult. Due to the nonlinear, anisotropic, and viscoelastic properties of dental and maxillofacial tissues, the finite element analysis process is highly complex. Studies have shown that when deformations are small, material properties can be simplified and assumed to be continuous, homogeneous, and isotropic linear elastic materials (Hemanth et al., 2015a; Hemanth et al., 2015b). In this study, we adopted a simplified material property setting, even though the deformations were small, which may result in some degree of information loss and potentially affect the geometric and mechanical properties of the model. Furthermore, in clinical practice, patients have different dietary habits, brushing methods, chewing preferences, and frequencies of aligner removal, leading to non-uniform wear of attachments. In this study, we selected the average wear amount and established the worn attachment models based on the primary wear patterns observed. This approach still has some discrepancies compared to real clinical conditions, and more accurate results would require analysis of a larger sample size collected from clinical settings. Additionally, in our previous clinical study, we used the most commonly used composite resin (Filtek Z350, 3M) for attachment fabrication to closely align with clinical practice. In this finite element study, we defined the material properties of the attachments as composite resin. However, different attachment materials, such as bioceramics, would exhibit different wear patterns and impacts on canine distalization. Therefore, the results of this study cannot be generalized to all attachment materials. More comprehensive results will require further research and exploration.

In addition to the aforementioned limitations, there is another significant issue to consider. The optimized attachments are tools designed and automatically added to the teeth by a computer on the basis of treatment goals, tooth position, and the spatial arrangement of the crown surfaces to assist clear aligners in achieving various complex tooth movements. Each optimized attachment has an active force application surface that provides force in a specific direction. The ability of optimized attachments to apply active force is attributed to the slight discrepancies between the final produced aligner and the active force application surface of the attachment. The aligner exerts pressure on the force application surface of the attachment to generate active orthodontic forces, and the magnitude of these forces is determined by the extent of the aforementioned discrepancies. In previous three-dimensional finite element studies involving optimized attachments, there has been no consensus on whether additional forces should be applied to the active force application surfaces of the attachments and, if so, the magnitude of these forces (Goto et al., 2017; Kim et al., 2020). This is because the actual forces applied by optimized attachments vary depending on the specific tooth conditions and decrease dynamically as the attachments wear, making accurate simulation of the clinical reality difficult. Therefore, in this study, no additional loads were applied to the active force application surfaces of the optimized root control attachments. Considering that the aligner actively applies force to the active force application surfaces of the optimized root control attachments, the clinical tendency for the canines to move in a tipping manner may be less pronounced than the results of this study, but the distal displacement of the canine crowns should not exceed the findings of this study. Thus, this study provides only a theoretical reference for the impact of optimized root control attachment wear on canine distalization.

Since clinical trials provide the highest level of evidence, the results of this study need to be validated through corresponding real-world studies. However, it is challenging to clinically validate the impact of attachment wear on tooth movement because numerous factors can influence the efficiency of tooth movement during clear aligner treatment in clinical practice. These factors include patient compliance with aligner wear, the rationality of the treatment plan, the material properties of the aligners, and the fit between the aligners and the teeth. It is difficult to attribute the efficiency of tooth movement solely to attachment wear. Furthermore, during the treatment process, teeth move in three-dimensional directions, and different types of tooth movements occur at different stages of treatment. For example, canine distalization is not a continuous process throughout the entire treatment period. Therefore, it is challenging to isolate the specific impact of attachments on a particular type of tooth movement. To achieve clinical validation, future studies will need to be specially designed to address this issue.

## 5 Conclusion

Given the limitations of the study, the following conclusions can be drawn:

1) Regardless of the type of attachment added to the canine or the amount of attachment wear, the canine tended to tip when maxillary canine distalization was performed with clear aligners.
2) As the degree of wear of the attachments increased, the efficiency of maxillary canine distalization decreased. The impact of wear on the rectangular attachments was relatively slight. Even with wear, the addition of rectangular attachments can achieve greater efficiency in maxillary canine distalization than optimized root control attachments.
3) Once optimized root control attachments have been in place for more than 4 months and maxillary canine distalization is still required, orthodontists should pay particular attention to the wear of these attachments to ensure the efficiency of canine distalization. If necessary, timely restoration of the attachment shape or rebonding of the attachments is recommended.

## Data Availability

The original contributions presented in the study are included in the article/Supplementary Material, further inquiries can be directed to the corresponding author.
