# Supplementary material for: Impacts of surface wear of attachments on maxillary canine distalization with clear aligners: a three-dimensional finite element study
Source: Front Bioeng Biotechnol. 2025 Jan 21;13:1530133. doi: 10.3389/fbioe.2025.1530133 (PMC11790557; doi:10.3389/fbioe.2025.1530133)
Supplement: Supplementary file 1 [file Table1.docx]

***Supplementary Material***

**1 Supplementary Tables**

**Supplementary Table 1.** The displacement, rate of change, and efficiency of distalization of the canine crown.

| Modes | Displacement (mm) | | Rate of change (%) | | Efficiency (%) | |
| --- | --- | --- | --- | --- | --- | --- |
|  | Model #1 | Model #2 | Model #1 | Model #2 | Model #1 | Model #2 |
| M0 | 0.1462 | 0.1277 | — | — | 73.10% | 63.85% |
| M2 | 0.1405 | 0.1237 | 3.90% | 3.13% | 70.25% | 61.85% |
| M4 | 0.1386 | 0.1206 | 5.20% | 5.56% | 69.30% | 60.30% |
| M6 | 0.1382 | 0.1001 | 5.47% | 21.61% | 69.10% | 50.05% |
| M8 | 0.1286 | 0.0958 | 12.04% | 24.98% | 64.30% | 47.90% |

Model #1: rectangular attachment group; Model #2: optimized root control attachment group.

**Supplementary Table 2.** The amount and rate of change of the canine root equivalent stress.

| Modes | Von mises stress (MPa) | | Rate of change (%) | |
| --- | --- | --- | --- | --- |
|  | Model #1 | Model #2 | Model #1 | Model #2 |
| M0 | 4.429 | 3.234 | — | — |
| M2 | 4.403 | 3.018 | 8.72% | 6.68% |
| M4 | 3.888 | 2.813 | 12.21% | 13.02% |
| M6 | 3.650 | 2.133 | 17.59% | 34.04% |
| M8 | 3.065 | 2.112 | 30.80% | 34.69% |

Model #1: rectangular attachment group; Model #2: optimized root control attachment group.

**Supplementary Table 3.** The amount and rate of change of the canine PDL equivalent stress.

| Modes | Von mises stress (MPa) | | Rate of change (%) | |
| --- | --- | --- | --- | --- |
|  | Model #1 | Model #2 | Model #1 | Model #2 |
| M0 | 0.176 | 0.141 | — | — |
| M2 | 0.169 | 0.136 | 3.98% | 3.55% |
| M4 | 0.165 | 0.130 | 6.25% | 7.80% |
| M6 | 0.158 | 0.119 | 10.23% | 15.60% |
| M8 | 0.147 | 0.114 | 16.48% | 19.15% |

PDL: Periodontal ligament; Model #1: rectangular attachment group; Model #2: optimized root control attachment group.
